# Establishment and Evaluation of Exosomes-Related Gene Risk Model in Hepatocellular Carcinoma

**DOI:** 10.1007/s10528-023-10441-6

**Published:** 2023-07-05

**Authors:** Lin Zhu, Yan Lou, Qiyu Xiao, Ling Wang, Guodong Chen, Wenjun Yang, Tengjiao Wang

**Affiliations:** 1 Key Laboratory of Fertility Preservation and Maintenance, The School of Basic Medicine, The General Hospital, Ningxia Medical University, Yinchuan, Ningxia China; 2 Department of Orthopedic Oncology, Spine Tumor Center, Changzheng Hospital, Naval Military Medical University, Shanghai, China; 3 Department of Nuclear Medicine, Hunan Cancer Hospital/The Affiliated Cancer Hospital of Xiangya School of Medicine, Central South University, Changsha, China; 4 Department of Stem Cells and Regenerative Medicine, Center for Translational Medicine, Naval Medical University, Shanghai, China; 5 Shanghai Key Laboratory of Cell Engineering, Shanghai, China; 6 Shanghai Institute of Stem Cell Research and Clinical Translation, Shanghai, China; 7 Department of Emergency, The Sixth Medical Center of PLA General Hospital, Beijing, China; 8 Department of Precision Medicine, Translational Medicine Research Center, Naval Medical University, Shanghai, China

**Keywords:** Hepatocellular carcinoma, Prognosis, Gene set enrichment analysis, Exosomes, Risk model

## Abstract

Hepatocellular carcinoma (HCC) is a challenging disease to evaluate in terms of prognosis, requiring close attention to the prognosis of HCC patients. Exosomes have been shown to play an important role in HCC development and have significant potential in managing HCC patient prognosis, as they are detectable in patients’ blood. By using small extracellular vesicular RNA, liquid biopsies can reflect the underlying physiological and pathological status of the originating cells, providing a valuable assessment of human health. No study has explored the diagnostic value of mRNA expression changes in exosomes for liver cancer. The present study investigated establishing a risk prognosis model based on mRNA expression levels in exosomes from blood samples of liver cancer patients and evaluated its diagnostic and prognostic value, providing new targets for liver cancer detection. We obtained mRNA data from HCC patients and normal controls from the TCGA and exoRBase 2.0 databases and established a risk prognostic assessment model using exosomes-related risk genes selected through prognostic analysis and Lasso Cox analysis. The patients were divided into high-risk and low-risk groups based on median risk score values to validate the independence and evaluability of the risk score. The clinical value of the model was further analyzed using a nomograph model, and the efficacy of immunotherapy and cell-origin types of prognostic risk genes were further assessed in the high- and low-risk groups by immune checkpoint and single-cell sequencing. A total of 44 genes were found to be significantly associated with the prognosis of HCC patients. From this group, we selected six genes (*CLEC3B*, *CYP2C9*, *GNA14*, *NQO1*, *NT5DC2*, and *S100A9*) as exosomal risk genes and used them as a basis for the risk prognosis model. The clinical information of HCC patients from the TCGA and ICGC databases demonstrated that the risk prognostic score of the model established in this study was an independent prognostic factor with good robustness. When pathological stage and risk prognostic score were incorporated into the model to predict clinical outcomes, the nomograph model had the best clinical benefit. Furthermore, immune checkpoint assays and single-cell sequencing analysis suggested that exosomal risk genes were derived from different cell types and that immunotherapy in the high-risk groups could be beneficial. Our study demonstrated that the prognostic scoring model based on exosomal mRNA was highly effective. The six genes selected using the scoring model have been previously reported to be associated with the occurrence and development of liver cancer. However, this study is the first to confirm that these related genes existed in the blood exosomes, which could be used for liquid biopsy of patients with liver cancer, thereby avoiding the need for puncture diagnosis. This approach has a high value in clinical application. Through single-cell sequencing, we found that the six genes in the risk model originate from multiple cell types. This finding suggests that the exosomal characteristic molecules secreted by different types of cells in the microenvironment of liver cancer may serve as diagnostic markers.

## Background

Hepatocellular carcinoma (HCC) is a multifaceted pathological process caused by various factors, including hepatitis B virus, hepatitis C virus, metabolic disorders, and alcoholism. Unfortunately, most HCC patients are initially diagnosed with advanced HCC, making it one of the deadliest cancers worldwide (Bray et al. 2018). Although there are various treatments for liver cancer, such as surgery, radiotherapy, and chemotherapy, the survival time of most liver cancer patients is still short, and prognostic testing for the disease has been emphasized (Forner et al. 2018). A hepatologic biopsy can effectively analyze the occurrence and progression of HCC and provide a diagnostic basis for the prognostic evaluation of patients (Wang et al. 2019). However, this procedure is not a routine practice because of the invasiveness of HCC biopsy, and the evaluation of HCC patient prognosis through non-invasive liquid biopsy could play a critical role in clinical management (Ahn et al. 2021).

In recent years, various liquid biopsy techniques have shown significant promise as prognostic tools for HCC. The detection of circulating tumor cells, cell-free DNA, and exosomes from blood samples solve the lack of key molecular targets for HCC patient prognostic testing (Chen et al. 2020b). Exosomes are 50–200-nm lipid vesicles containing nucleic acids, proteins, small metabolic molecules, and many other components. Studies have demonstrated that exosomes can enter cells through receptor-ligand interactions or through various mechanisms such as phagocytosis, endocytosis, micropinocytosis, and fusion, to activate signaling pathways among liver cells, different types of immune cells such as macrophages, natural killer cells, and stroma, and various stromal cells such as stellate cells and adipocytes (Shen et al. 2017; Liu and Li 2018; Zhou et al. 2018; Zhang et al. 2019, p. 7). Researchers have found that miR-21 levels in exosomes of HCC patients were significantly higher than in patients with chronic hepatitis and healthy controls and that exosomes were significantly more sensitive to detection based on patient serum, suggesting that specific molecules in exosomes could be a promising new marker for HCC (Wang et al. 2014). Furthermore, exosomal alterations reflect the prognosis and treatment of HCC. High expression of miRNA-21 and lncRNA-ATB in the exosomes suggests poor overall survival for HCC patients, indicating that different molecules in the exosomes of HCC patients reflect different pathophysiologic processes of the disease (Lee et al. 2019). Additionally, miR-122 expression was significantly reduced in the exosomes of patients treated with transarterial chemoembolization, further supporting the potential of exosomes as diagnostic markers for HCC (Suehiro et al. 2018).

Currently, most molecules targeted by exosomes for diagnosing HCC patients are small RNAs, such as miRNAs (Li et al. 2019). However, no studies have been reported to assess patient prognosis based on changes in mRNA expression levels in HCC exosomes, much less relevant risk prognostic models. However, the diagnostic value of mRNA in exosomes has not received sufficient attention. Liquid biopsy techniques based on small extracellular vesicular RNA are gradually entering clinical applications. Exosome Diagnostics is one company that developed ExoDx for blood testing based on *EML4-ALK* fusion transcripts and ExoDxTM Prostate (IntelliScore) for multiple gene expression features (*ERG*, PCA3, and *SPDEF*) in small extracellular vesicles. This study proposed three scientific questions regarding the detection of mRNA in the secretions of liver cancer patients for prognosis. First, are there any mRNA molecules whose differential expression characteristics are shared by liver cancer tissue and peripheral blood secretions of liver cancer patients? Second, can relevant mRNA molecules be used to evaluate the prognosis of liver cancer patients? Finally, which cell types of liver cancer tissue are the source of relevant mRNA molecules? These questions must be answered to determine the diagnostic value of exosomal mRNA in liver cancer patients.

We aimed to fill this gap by screening key mRNAs in the exosomes of HCC patients to establish a risk prognostic model for HCC patients. The risk prognostic risk score obtained through the model as an independent risk factor can more accurately assess the 1-, 3-, and 5-year survival rates of patients and has a high application value.

## Materials and Methods

### Data Collection

Exosome transcriptome data from healthy human blood and liver cancer patients were obtained from the exoRBase 2.0 database (http://www.exorbase.org/exoRBaseV2/toIndex). mRNA expression data and copy number variation data from liver cancer (https://xena.ucsc.edu/) were obtained from the TCGA database via the ICGC website ( https://dcc.icgc.org/) downloaded transcriptome data of liver cancer. Data from 374 tumor samples and 50 control samples were downloaded from the TCGA database, whereas data from 112 tumor samples and 118 control samples were downloaded from exoRBase 2.0. mRNA expression values were standardized and converted to log2 (TPM + 1), with copy number amplification and deletion thresholds determined using the GISTIC software.

## Differentially Expressed Genes and Exosomes Prognostic Genes (Exo-Genes) Screening

HCC and control data from the TCGA and exoRBase 2.0 databases were compared using the limma package. |LogFC|> 0.585 and Calibration *P* < 0.05 were defined as differentially expressed genes (DEGs). Finally, 123 overlapping exosomes-related differential genes were filtered by intersecting the differential genes from the two databases. To further explore the clinical guidance value of the differential genes, we matched the exosomes-related differential genes from the TCGA database with clinical data and then used the survival package to evaluate the prognostic difference through univariate Cox regression analysis followed by the log-rank test, and the exosomes-related differential genes with a P < 0.05 were defined as exosomes prognostic genes (Exo-genes). The ability of related genes to influence patient prognosis was validated by plotting Kaplan Meier survival curves of exosomes prognostic genes.

## Establishment and Validation of Risk Prognosis Model

The gene expression data of Exo-genes were combined with clinical data, and prognostic models were constructed using the glmnet package with Lasso-Penalized Cox regression analysis to exclude genes predisposed for fitting. A risk prognostic model was constructed in the TCGA database of HCC patients with LASSO regression analysis, and the obtained coefficients and gene expression values were used to derive a risk prognostic score, formulated as follows: Risk score = sum (Expgene × coef). Median liver cancer expression data from the TCGA database were categorized into high- and low-risk groups based on risk prognostic score. The prognostic model predictive capacity was assessed using Kaplan–Meier survival curves and receiver operating diagnostic (ROC) curve analysis. The model was repeated in liver cancer data (LIRI-JP) downloaded using ICGC to test the accuracy of the predictive ability of risk scores. To further investigate the robustness of risk prediction models to different clinical factors, we applied risk prognostic scores to different subgroups of clinical factors and mapped Kaplan–Meier survival curves for validation. Single and multivariable Cox regression analyses of risk prognostic scores and clinical variables in TCGA-LIHC and LIRI-JP revealed that risk prognostic scores were independent risk factors in both analyses (*P* < 0.05).

## Establishment of a Nomograph Model

The Rms package was used to develop a prognostic column model to provide more accurate prognostic predictions for clinical patients based on risk scores and clinical characteristics. Identification accuracy was estimated using the consistency index (c index), the area under the ROC curve (AUC), and calibration diagrams. The estimates c Index and AUC > 0.7 are reasonable. Decision curve analysis (DCA) was then used to evaluate the clinical utility of the nomogram model.

## Immune Infiltration Analysis

HCC expression data from the TCGA database were categorized into high- and low-risk groups based on median risk prognostic score. We calculated immune checkpoint expression in both groups and presented them with a point matrix. The ratio of 22 immune cell types per sample was calculated by cell-type identification with estimator relative subsets of RNA transcripts (CIBERSORT).

## Single-Cell Sequencing Analysis

GSE125449 was downloaded for analysis. Sequencing data were qualitatively controlled using Seurat packets, and the cells were clustered to analyze the expression of exosomal risk genes in different clusters. Different cluster cell types were analyzed using singleR packets for annotation.

## Statistical Analysis

The difference between the two groups was compared using the unpaired t-test. Single and multifactorial Cox regression analyses were used to analyze the relationship between the variables and the patient outcome. In all analyses, *P* < 0.05 was considered statistically significant.

## Results

### Screening of Exosome-Related DEGs in HCC

**Fig. 1:**
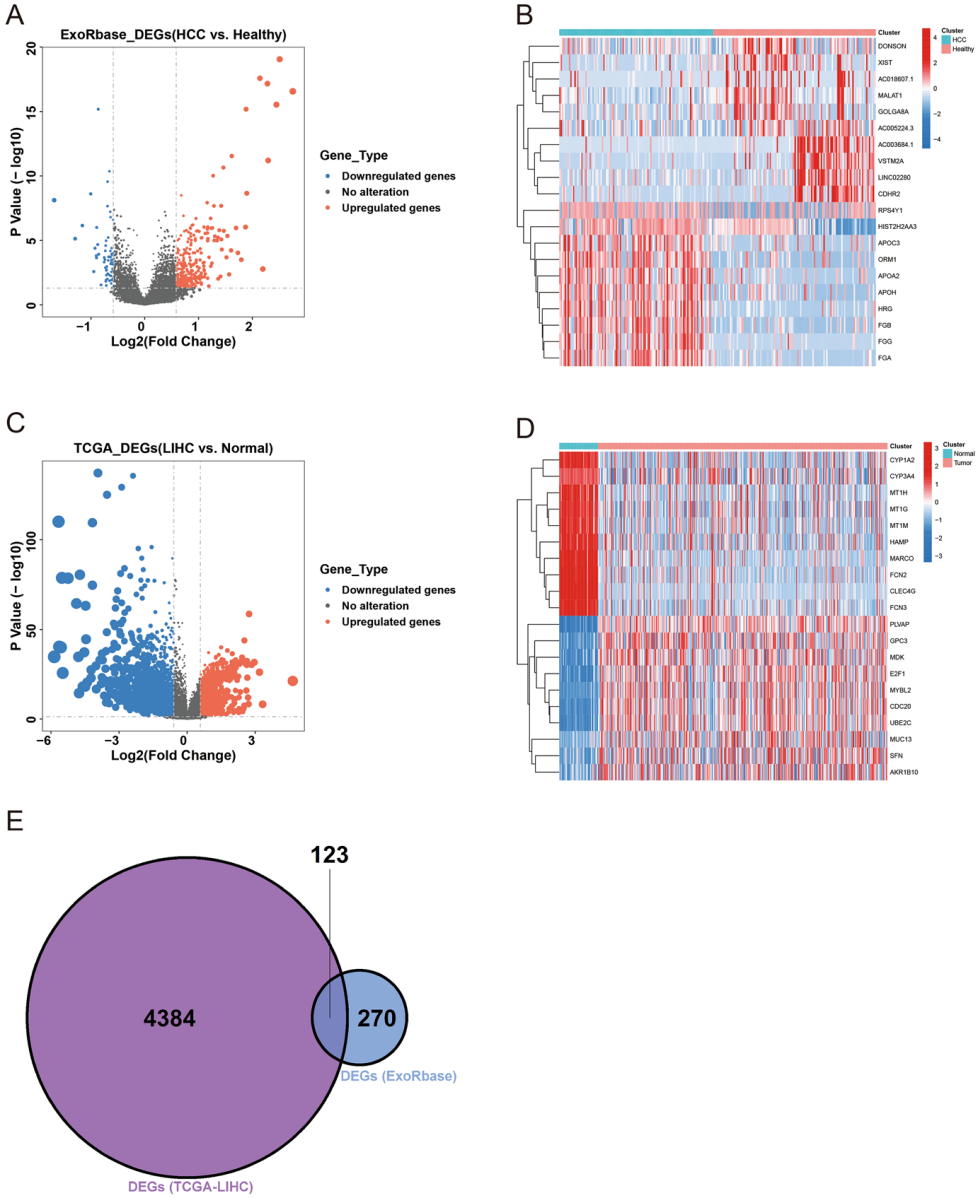
Screening of 123 HCC exosomes-related differential genes. **A** Volcano plot of differential gene expression in hepatocellular carcinoma in the exoRBase 2.0 database. **B** Top 20 differentially expressed hepatocellular carcinoma heat maps in exoRBase 2.0 database. **C** Volcano plot of differential gene expression in hepatocellular carcinoma in TCGA database. **D** Top 20 differentially expressed hepatocellular carcinoma heat maps in TCGA database. **E** Intersection of differentially expressed genes in liver cancer between exoRBase 2.0 database and TCGA database

We compared the differences in exosomes-related genes between HCC patients and normal controls in the exoRBase 2.0 database and found 393 differentially expressed genes (Fig. 1A). *DONSON*, *XIST*, *AC018607.1*, *MALAT1*, *GOLGA8A*, *AC005224.3*, *AC003684.1*, *VSTM2A*, *LINC02280*, and *CDHR2* were under-expressed in exosomes from liver cancer patients, and *RPS4Y1*, *HIST2H2AA3*, *APOC3*, *ORM1*, *APOA2*, *APOH*, *HRG*, *FGB*, *FGG*, *FGA* were highly expressed in the exosomes of HCC patients (Fig. 1B). We also compared genetic differences between HCC patients and normal controls in the TCGA database and found 4,507 differentially expressed genes (Fig. 1C), with thermal mapping revealing the top 20 most differentially expressed genes (Fig. 1D). A cross-section analysis of differentially expressed genes from the two databases resulted in 123 exosomal-associated differential genes in HCC (Fig. 1E).

## Identification of Exosomes Prognostic Genes (Exo-Genes)

**Fig. 2:**
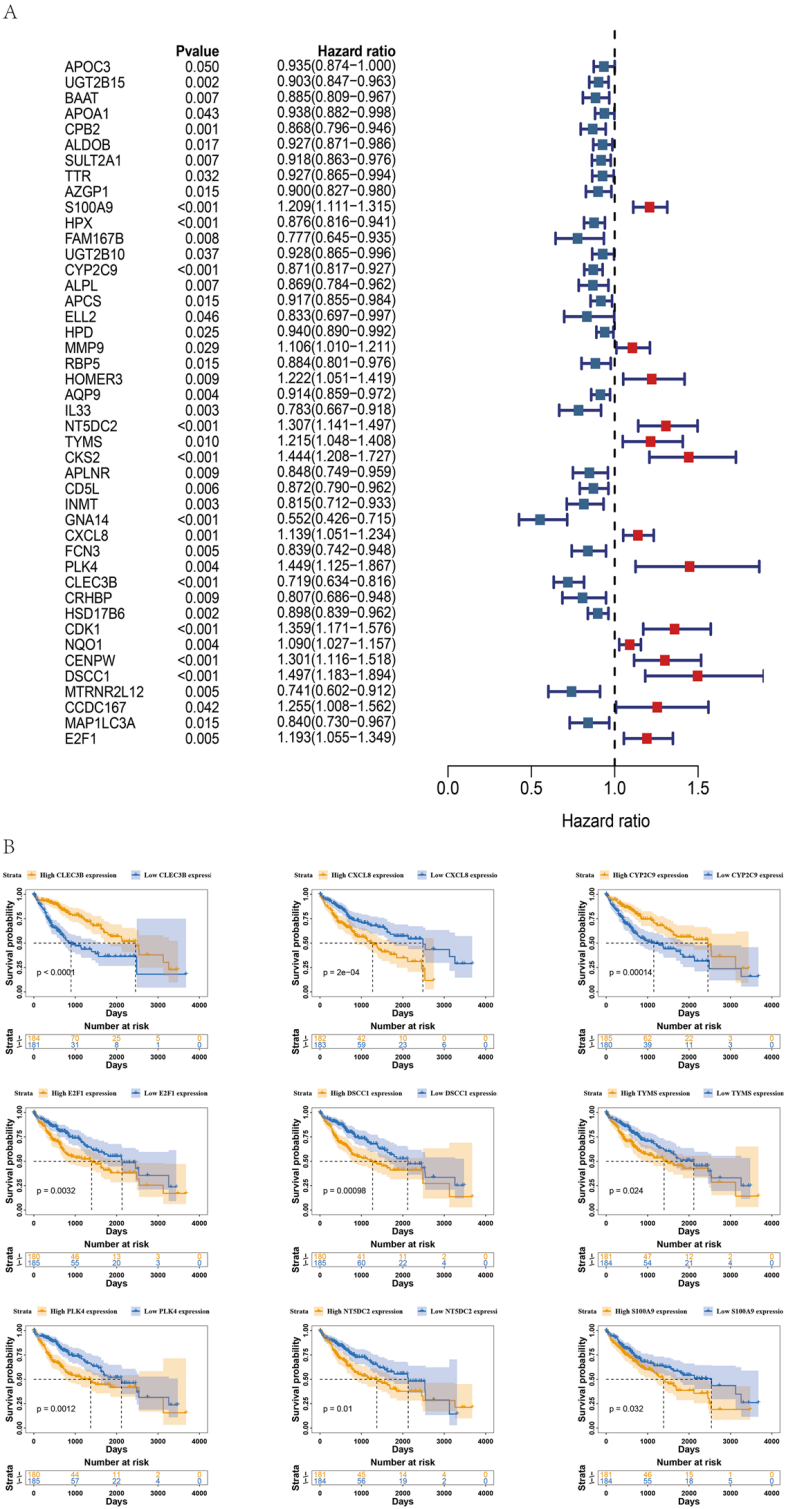
Identification of 44 exosomes prognostic genes (Exo-genes) in hepatocellular carcinoma. **A** Identification of exosomes prognostic genes by univariable Cox regression analysis. **B** Prognostic curves of high and low expression of exosomes prognostic genes from hepatocellular carcinoma patients in TCGA database

To further define the relationship between HCC exosomes-related differential genes and patient prognosis, we performed a univariate regression analysis of 123 HCC exosomes-related differential genes and identified 44 genes as Exo-genes. Of these, 14 genes were risk factors and 30 were protective (Fig. 2A). Based on the median expression of these prognostic genes, we divided the data into high- and low-expression groups, mapped Kaplan–Meier survival curves, and selected the top nine genes with the lowest *P* values for presentation (Fig. 2B). We confirmed that the patients with high expression of *DSCC1*, *CXCL8*, *S100A9*, *NT5DC2*, *E2F1*, *PLK4,* and *TYMS* had lower overall survival rates. The patients with high expression of *CLE3B* and *CYP2C9* exhibited higher overall survival rates.

## Establishment of Risk Prognostic Model Based on Exosomal Risk Genes

**Fig. 3:**
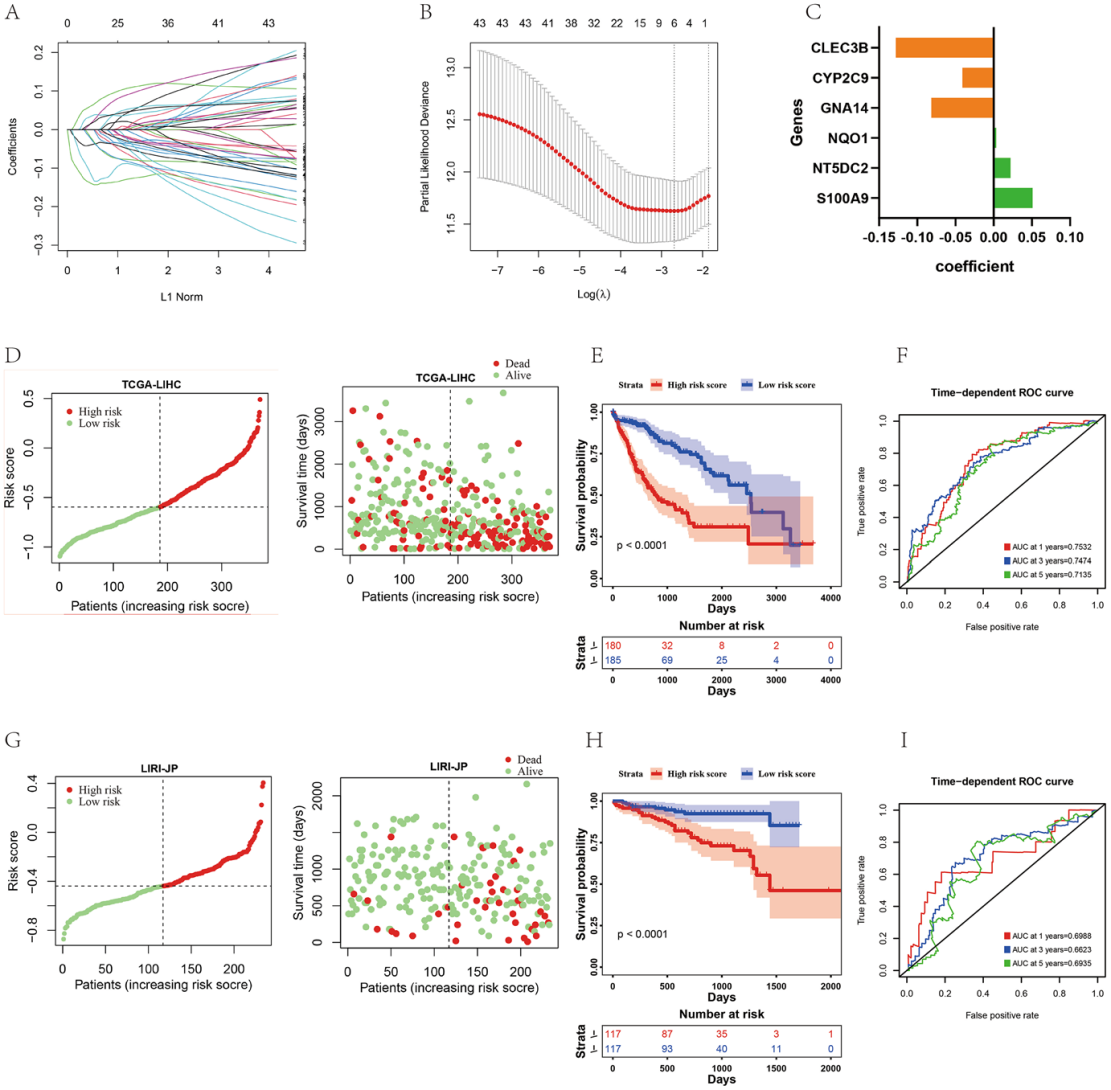
Risk prognosis model based on exosomal risk genes has good diagnostic value. **A** Changes in regression coefficient of exosomes prognostic genes in Lasso Cox regression analysis. **B** Determination of λ. **C** Regression coefficient of CLEC3B, CYP2C9, GNA14, NQO1, NT5DC2 and S100A9. **D** HCC patients with high-risk prognostic score in TCGA database have lower survival. **E** Survival curves of high-risk and low-risk prognostic score groups in TCGA database of HCC patients. **F** ROC validates prognostic performance of HCC risk score in TCGA database. **G** HCC patients with high-risk prognostic score in ICGC database have lower survival. **H** Survival curves of high-risk and low-risk prognostic groups of liver cancer patients in ICGC database. **I** ROC validates prognostic performance of HCC risk score in ICGC database

To further evaluate the prognostic power of exosomal prognostic genes in HCC patients, we performed the Lasso Cox regression analysis based on TCGA HCC patient prognostic data. Figure 3A demonstrates regression coefficient changes in each exosomal prognostic gene, with the model having the lowest mean variance when lambda is 0.0678. Consequently, the following six genes (exosomal risk genes) were selected to construct the risk prognostic model (Fig. 3B): *CLEC3B*, *CYP2C9*, *GNA14*, *NQO1*, *NT5DC2*, and *S100A9*. Based on the expression and regression coefficient (Fig. 3C) of these six genes, we constructed a risk prognosis score based on the following formula:

1 $${\text{Riskscore}} = \, {\text{S}}100{\text{A}}9 \times 0.0510 - {\text{CYP}}2{\text{C}}9 \times \, 0.0410 + {\text{NT5DC2}} \times 0.0222 - {\text{CLEC3B}} \times \, 0.1280 + {\text{NQO1}} \times 0.0035 - {\text{GNA14}} \times 0.0816$$

To explore the prognostic power of risk score for patient outcomes, we categorized HCC patients in the TCGA database (TCGA-LIHC) into high- and low-risk groups (Fig. 3D) based on median risk scores and found that overall survival was significantly lower in the high-risk group than in the low-risk group (Fig. 3E). Using AUC, we found that risk prognostic scores were accurately predictive in 1-, 3-, and 5-year survival assessments (1-year AUC = 0.7532, 3-year AUC = 0.7474, and 5-year AUC = 0.7135) (Fig. 3F). The same study was performed in validation data from the ICGC database (LIRI-JP), and similar results were obtained from TCGA-LIHC, suggesting that this risk model has broad applicability (Figs. 3G–I).

## Risk Prognostic Score is an Independent Prognostic Factor with Good Robustness

**Fig. 4:**
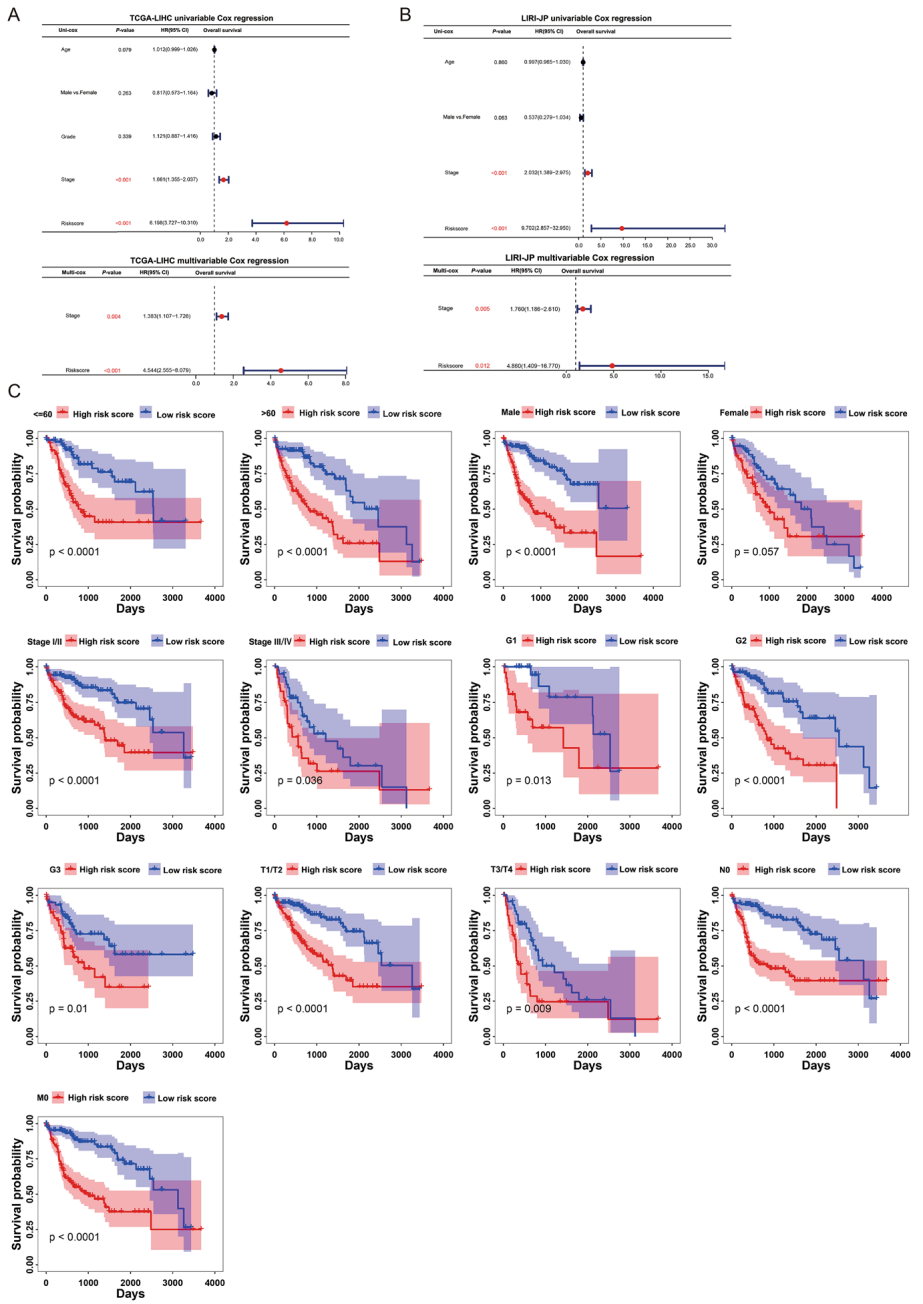
Risk prognostic score is an independent prognostic factor with good robustness. **A** Univariable cox regression analysis and multivariable cox regression analysis of survival of HCC patients in the TCGA database. **B** Univariable cox regression and multivariable cox regression analysis of HCC in ICGC Database. **C** HCC patients in the TCGA database were subgroups based on clinical characteristics and Kaplan–Meier demonstrated good predictive ability of risk prognosis scores

To explore the independent prognostic value of risk score, we performed univariable and multivariable Cox regression analyses with age, sex, stage, grade, and risk score of TCGA-LIHC data, indicating that risk score was an independent prognostic factor for patients in the TCGA-LIHC data (HR = 4.544, *P* < 0.001) (Fig. 4A). We also observed risk score as an independent prognostic factor for patients in the LIRI-JP data (HR = 4.860, *P* < 0.05) (Fig. 4B).

We evaluated the robustness of TCGA-LIHC based on identifying risk score as an independent predictor. By classifying the clinical characteristics (age, sex, stage, and grade) of TCGA-LIHC, we evaluated the outcomes of patients with different risk scores in different subgroups, suggesting that patients with higher risk scores had worse outcomes than those with lower risk scores, suggesting a more robust risk model (Fig. 4C).

## Evaluation of Nomogram Model for Risk Prognosis Score and Detection of Immune Checkpoint

**Fig. 5:**
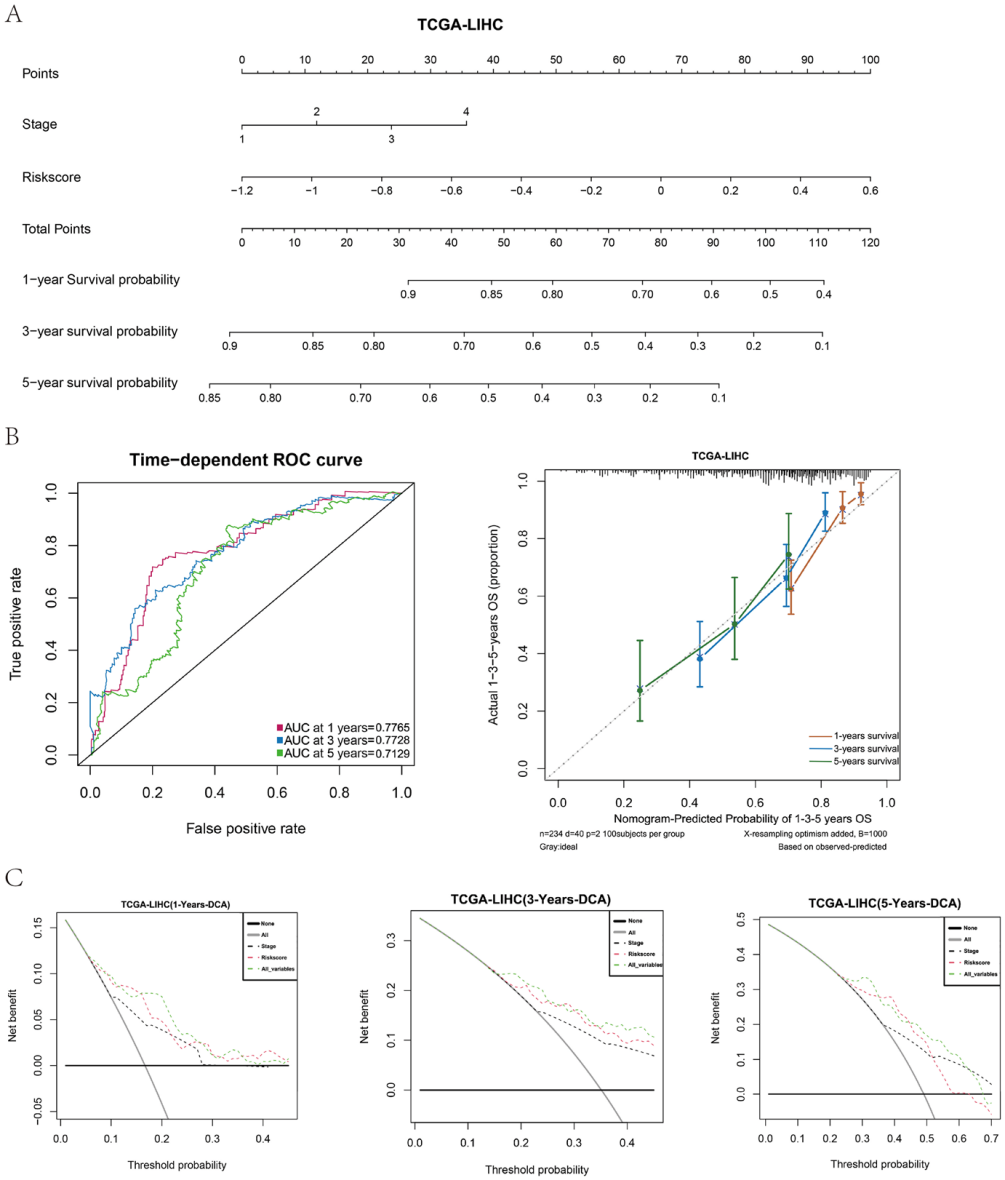
A nomogram model constructed based on pathological stage and risk prognostic score was able to effectively predict the survival of liver cancer patients in TCGA database. **A** Nomogram model based on HCC patients in TCGA database. **B** Time dependent ROC curve and calibration curve based on Nomogram model of liver cancer patients in TCGA database. **C** DCA curve of liver cancer patients based on TCGA database

We constructed a nomogram survival prediction model based on the pathological stages and risk prognostic scores of HCC patients to predict 1-, 3-, and 5-year survival probabilities (Fig. 5A) of TCGA-LIHC. The ROC and calibration curves revealed that the model had an accurate predictive ability for 1-, 3-, and 5-year survival probabilities of patients (Fig. 5B). In addition, the decision curve analysis revealed that the model exhibited the best clinical benefit (Fig. 5C) when incorporating pathologic stage and risk prognosis score to predict clinical outcomes.

**Fig. 6:**
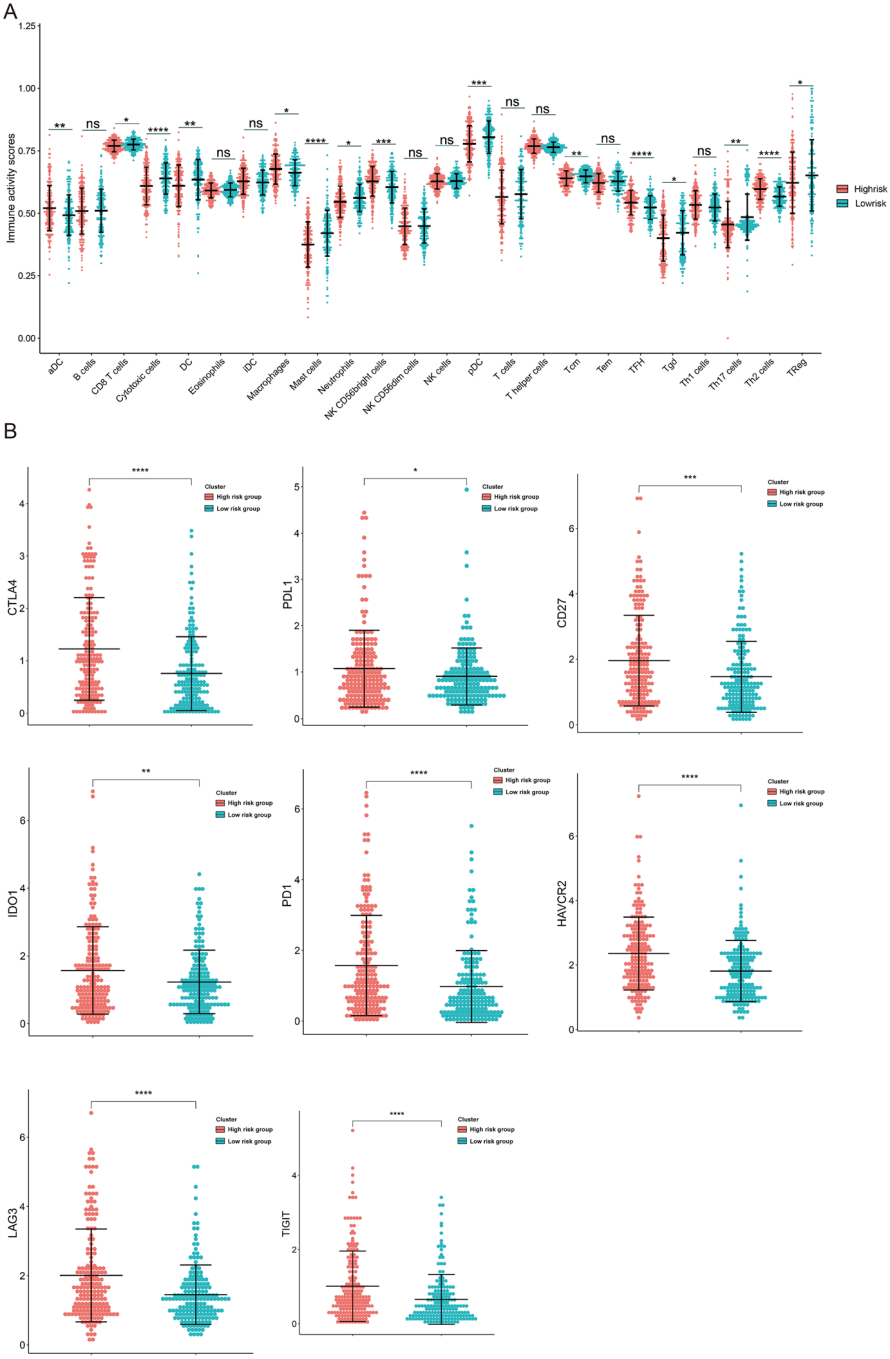
Differences in immune test sites among patients with different risk prognosis scores. **A** Difference of distribution of immune cells in high/low-risk groups of HCC patients in TCGA database. **B** Differential expression of immunological test sites in high/low-risk groups of HCC patients in TCGA database

We compared immune cell types between the high- and low-risk groups with risk prognostic scores and found differences in the distribution of multiple immune cells (Fig. 6A). To further assess whether risk prognostic scores can guide immunotherapy in patients, we examined the expression of immune checkpoints in the high- and low-risk groups. We found that the expression of *CTLA4*, *PDL1*, *CD27*, *ID01*, *PD1*, *HAVCR2*, *LAG3,* and *TIGIT* was significantly higher in the high-risk group than in the low-risk group, suggesting that immunotherapy may benefit patients in the high-risk group (Fig. 6B).

## Distribution of Exosomal Risk Genes in Hepatocellular Carcinoma

**Fig. 7:**
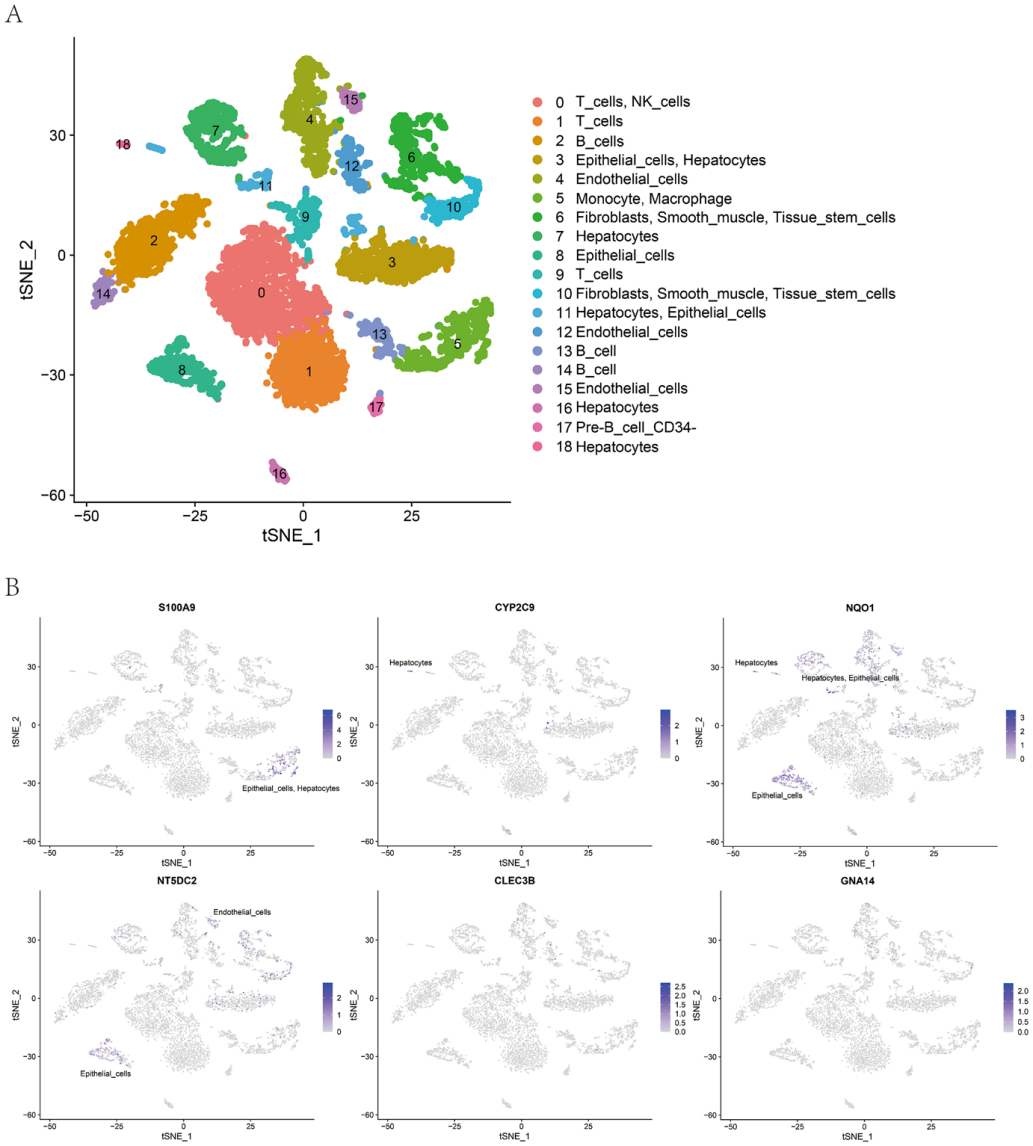
Distribution of six exosomal risk genes in HCC tissue. **A** Cell clustering for single-cell sequencing. **B** Distribution of six exosomal risk genes in different kinds of cells

Different cell types in the tumor microenvironment (e.g., fibroblasts, immune cells, endothelial cells, and hepatocytes) have broad implications for tumor progression and play a key role in regulating the bioactivity of HCC. To further understand the cell types expressed in HCC tissues by six exosomal risk genes (*CLEC3B*, *CYP2C9*, *GNA14*, *NQO1*, *NT5DC2*, and *S100A9*) that build risk prognostic models, we performed correlation analysis of single-cell sequencing dataset GSE125449. Through down-dimensional analysis, we identified 18 cell types in this single-cell sequencing dataset (Fig. 7A), with low expression of *CLEC3B* and *GNA14* in various cell types, high expression of *CYP2C9* in hepatocytes, high expression of *NQO1* in epithelial and hepatocytes, high expression of *NT5DC2* in epithelial and endothelial cells, and high expression of *S100A9* in multiple cell types, suggesting a role in HCC prognosis (Fig. 7B).

## Discussion

The tumor microenvironment plays a crucial role in tumor progression, and the interaction between different cells constitutes a significant component of the tumor microenvironment. Apart from direct cell-to-cell interactions, communication between cells can occur through the secretion of exosomes and other signaling molecules. Such communication within the tumor microenvironment can have a significant effect on tumor growth and metastasis (Wu et al. 2019). Exosomes—known to mediate the transfer of various molecules—play a pivotal role in the initiation, progression, invasion, and metastasis of hepatocellular carcinoma (HCC). Consequently, exosomes represent promising targets for developing diagnostic, therapeutic, and prognostic approaches for HCC. Understanding the mechanisms underlying exosomes-mediated communication within the tumor microenvironment may lead to developing novel strategies for effectively managing HCC (Abudoureyimu et al. 2019).

The evaluation of risk score models for tumor prognosis is focused on specific pathophysiological processes or signaling pathways involved in tumor progression, such as autophagy, aging, and iron death. However, the practical application of these models in clinical settings is limited by the difficulty of obtaining pathologic tissue samples and the limitations of relevant risk scores (Liang et al. 2020; Qiu et al. 2020; Chen et al. 2022). Exosomes in various biological fluids, such as serum, urine, and saliva, offer a simpler environment than complex tissue and cellular environments. Because of the relative stability of blood molecules, liquid biopsy is increasingly emphasized as a potential marker for diagnosing hepatocellular carcinoma progression and metastasis (Halvaei et al. 2018). Only one study evaluated exosomal mRNA prognostic risk models in triple-negative breast cancer. In the study, risk score models were established using *IVL*, *CXCL13*, and *AP2S1* genes. The results demonstrated that survival rates were significantly higher in low-risk groups than in high-risk groups (Qiu et al. 2021).

Our study demonstrated that 44 mRNAs present in exosomes were associated with the prognosis of HCC patients. Among them, *CLEC3B*, *CYP2C9*, *GNA14*, *NQO1*, *NT5DC2*, and *S100A9* were selected as exosomal risk genes to construct a risk prognosis model. This model accurately predicted the prognosis of patients as an independent prognostic factor, confirming the clinical value of mRNAs present in patients’ exosomes.

Of the six genes in our risk model, *CLEC3B*, *CYP2C9*, and *GNA14* were identified as protective genes for patients’ prognoses. Specifically, *CLEC3B* exhibited a protective effect. Previous studies have demonstrated that decreased *CLEC3B* expression indicates poor prognosis in liver cancer patients, possibly because it regulates immune cell infiltration in liver cancer tissue (Xie et al. 2020). Moreover, studies (Dai et al. 2019) have confirmed that liver cancer cells with decreased *CLEC3B* expression secrete substances that promote tumor cell and endothelial cell invasion and migration while inhibiting angiogenesis. These effects ultimately worsen patients’ prognosis. Furthermore, studies (Bodin et al. 2005) have confirmed the protective effect of *CYP2C9* on liver cancer prognosis. For instance, studies have analyzed differentially expressed genes from multiple liver cancer datasets in the GEO database and identified eight hub genes correlating with survival rates in liver cancer patients. Notably, *CYP2C9* was identified as one of the key genes in these analyses. The synthesis of the *CYP2C9* gene is often associated with metabolism. Consequently, some researchers have examined the relationship between metabolism-related genes, lipid metabolism-related genes, and *CYP2C* subfamily genes and the prognosis of liver cancer patients. These studies have confirmed that *CYP2C9* is an independent prognostic factor with high diagnostic value (Wang et al. 2018; Huo et al. 2021; Xu et al. 2022). These studies have performed differential gene analysis on liver cancer data from multiple datasets of the TCGA database and GEO. These studies have identified *GNA14* as one of the key characteristic genes that affect the prognosis of liver cancer patients (Yu et al. 2020a; Ye et al. 2022). Subsequent experiments have confirmed the discovery that *GNA14* is a prognostic protective gene in patients with liver cancer. The downregulation of *GNA14* in hepatocellular carcinoma is significantly associated with tumor grade, clinical stage, and T stage. Knocking out *GNA14* in cells can significantly promote cancer growth, and the related mechanism is linked to the activation of signaling pathways such as MAPK/JNK, PI3K/AKT, and Notch1 (Yu et al. 2020b; Song et al. 2021; Xu et al. 2021).

The risk model for liver cancer patients includes *NQO1*, *NT5DC2*, and *S100A9* as risk genes. Several studies (Lin et al. 2017; Zhao et al. 2021) have confirmed that *NQO1* mRNA levels in liver cancer tissues are significantly upregulated compared with that in non-liver cancer tissues. The high expression of *NQO1* is associated with tumor size and degree of venous infiltration of hepatocellular carcinoma, making it a potential biomarker for prognostic evaluation of hepatocellular carcinoma patients. Functional experiments have revealed that *NQO1* not only activates the transcriptional activity of SREBP1 to promote the progression and metastasis of liver cancer but also promotes the growth and invasion of liver cancer via the ERK/p38-NRF2 signaling pathway. These findings indicate that *NQO1* may play a crucial role in the development and progression of liver cancer (Yang et al. 2021; Wang et al. 2022). Most members of the NT5DC family are highly expressed in liver cancer, and specific studies (Lin et al. 2017; Zhao et al. 2021) have confirmed that elevated expression of *NT5DC2* is linked to higher liver cancer stages and a poorer prognosis for patients. These findings suggest that *NT5DC2* may be a potential prognostic marker for liver cancer (Chen et al. 2020a; Li et al. 2022). Moreover, functional experiments have validated that the overexpression of *NT5DC2* can impede EGFR ubiquitination, leading to the promotion of HCC cell proliferation and cloning in vitro, as well as the stimulation of tumor growth in vivo. These results suggest that *NT5DC2* may play a vital role in the development and progression of liver cancer through the EGFR signaling pathway (Li et al. 2020). This study has confirmed *S100A9* as a prognostic risk gene in patients, and high levels of *S100A9* in the serum indicate a poor prognosis. Previous studies have reported a significant increase in the expression of *S100A9* in the tissues and serum of liver cancer patients. These findings suggest that *S100A9* may serve as a potential prognostic marker for liver cancer and could be a target for therapeutic interventions (Duan et al. 2018; Meng et al. 2019). Furthermore, functional experiments (Wu et al. 2015) have validated that upregulation of S100A9 can bind to RAGE and activate the RAGE-dependent MAPK signal cascade, promoting cell growth and invasion in HCC. These results suggest that *S100A9* may play a crucial role in the progression of liver cancer by activating the RAGE signaling pathway.

No research has explored the diagnostic value of mRNA expression changes in exosomes for liver cancer. The present study investigated establishing a risk prognosis model based on mRNA expression levels in exosomes from blood samples of liver cancer patients and evaluated its diagnostic and prognostic value, providing new targets for liver cancer detection. As almost all types of cells are capable of secreting exosomes, it is possible that exosomes from blood cells, endothelial cells, and various organ cells may be present in the blood. Therefore, the genes found in peripheral blood exosomes may also be influenced by cells outside of liver cancer tissue. To address this issue, further research should be conducted in two areas. Firstly, the expression levels of risk genes in liver cancer cells should be altered to evaluate their impact on the changes in risk gene expression in peripheral blood exosomes. Secondly, single extracellular vesicle detection technology should be employed to screen for exosomes with liver cancer-specific markers, in order to ensure that the risk genes selected are secreted from liver cancer tissue. Further research will be conducted in these two areas to clarify the correlation between exosomal risk genes and liver cancer-related genes.

This study is the first to validate that mRNA molecules present in peripheral blood secretions could be utilized to predict the prognosis of liver cancer patients, thereby offering new targets and avenues for the prognosis diagnosis of liver cancer patients. These findings may have significant clinical implications in developing non-invasive prognostic tests for liver cancer. Through single-cell sequencing analysis, we discovered that the six genes in the risk model were derived from multiple cell types, indicating that various cells in the microenvironment of liver cancer tissue may contribute to the occurrence and development of liver cancer. Our findings suggest that exosomes derived from different cell types in the blood may have prognostic value for liver cancer.

## Conclusion

In this innovative comparative research, we identified 44 exosomal prognostic genes from the genomes of Chinese and foreign HCC patients and normal controls. We performed functional enrichment and copy number analysis on these genes and selected six genes—*CLEC3B*, *CYP2C9*, *GNA14*, *NQO1*, *NT5DC2*, and *S100A9*—as exosomal risk genes through Lasso Cox regression analysis. These genes were used to establish risk prognosis models validated in TCGA and ICGC databases. Our study used Cox regression analysis, robustness analysis, column model evaluation, immune checkpoint testing, and single-cell sequencing to confirm the utility of the risk prognostic score.
